# Interdisciplinary Delphi study by PROSEC North America: Recommendations on single indirect restorations made from ceramic and nonmetallic biomaterials for posterior teeth

**DOI:** 10.1111/jerd.13289

**Published:** 2024-08-02

**Authors:** Florin Eggmann, Amelia L. Orta, Awab Abdulmajeed, Wael Att, Florian Beuer, Michael Bergler, Markus B. Blatz, Jakob Brief, Victor E. Castro, Lyndon F. Cooper, Sean Han, Miguel A. Ortiz, Rade D. Paravina, Peter Pizzi, Michael J. Tholey, Julián Conejo

**Affiliations:** ^1^ Department of Preventive and Restorative Sciences, Robert Schattner Center, Penn Dental Medicine University of Pennsylvania Philadelphia Pennsylvania USA; ^2^ Department of Periodontology, Endodontology, and Cariology University Center for Dental Medicine Basel UZB, University of Basel Basel Switzerland; ^3^ Department of Advanced Oral Sciences and Therapeutics, Division of Prosthodontics University of Maryland School of Dentistry, University of Maryland Baltimore Maryland USA; ^4^ Department of Restorative Sciences, Advanced Education in Prosthodontics The Dental College of Georgia at Augusta University Augusta Georgia USA; ^5^ Department of General Practice and Prosthodontics, School of Dentistry Virginia Commonwealth University Richmond Virginia USA; ^6^ Department of Prosthetic Dentistry, Center for Dental Medicine Medical Center‐University of Freiburg, Faculty of Medicine, University of Freiburg Freiburg Germany; ^7^ Department of Prosthodontics, Geriatric Dentistry and Craniomandibular Disorders Charité‐Universitätsmedizin Berlin, Corporate Member of Freie Universität Berlin and Humboldt Universität zu Berlin Berlin Germany; ^8^ PROSEC gGmbH Bad Säckingen Germany; ^9^ VITA Zahnfabrik Bad Säckingen Germany; ^10^ Studio‐280 Houston Texas USA; ^11^ School of Dentistry Virginia Commonwealth University Richmond Virginia USA; ^12^ Master's Arch Phoenix Arizona USA; ^13^ Department of Restorative Dentistry University of Illinois College of Dentistry Chicago Illinois USA; ^14^ Department of Restorative Dentistry and Prosthodontics, John M. Powers, PhD, Center for Biomaterials and Biomimetics The University of Texas School of Dentistry at Houston Houston Texas USA; ^15^ Pizzi Dental Studio Staten Island New York USA

**Keywords:** crowns, Delphi technique, dental materials, inlays, onlays, permanent restoration

## Abstract

**Objective:**

This article puts forward consensus recommendations from PROSEC North America regarding single indirect restorations made from ceramic and nonmetallic biomaterials in posterior teeth.

**Overview:**

The consensus process involved a multidisciplinary panel and three consensus workshops. A systematic literature review was conducted across five databases to gather evidence. The recommendations, informed by findings from systematic reviews and formulated based on a two‐phase e‐Delphi survey, emphasize a comprehensive treatment strategy that includes noninvasive measures alongside restorative interventions for managing dental caries and tooth wear. The recommendations advocate for selecting between direct and indirect restorations on a case‐by‐case basis, favoring inlays and onlays over crowns to align with minimally invasive dentistry principles. The recommendations highlight the critical role of selecting restorative biomaterials based on clinical performance, esthetic properties, and adherence to manufacturer guidelines. They emphasize the importance of precision in restorative procedures, including tooth preparation, impression taking, contamination control, and luting. Regular follow‐up and maintenance tailored to individual patient needs are crucial for the longevity of ceramic and nonmetallic restorations.

**Conclusions:**

These PROSEC recommendations provide a framework for dental practitioners to deliver high‐quality restorative care, advocating for personalized treatment planning and minimally invasive approaches to optimize oral health outcomes.

**Clinical Significance:**

The PROSEC North America recommendations highlight the importance of minimally invasive techniques in posterior tooth restorations using ceramic and non‐metallic biomaterials. These principles prioritize tooth structure conservation and personalized treatment planning, essential for enhancing clinical outcomes and long‐term oral health.

## 1 INTRODUCTION

Dental caries continues to impose a heavy health burden worldwide, necessitating effective prevention and management strategies.^1^ Noninvasive measures are paramount to preventing and controlling carious lesions.^2^, ^3^ These measures include dietary control, mainly aimed at reducing the consumption of free sugars; biofilm control through twice or thrice daily tooth brushing; and mineralization control, primarily achieved via the topical application of fluorides.^2^, ^3^, ^4^, ^5^ While such noninvasive measures are essential in managing individual caries risk, carious lesions with cavitation and defective restorations that preclude adequate biofilm control often require restorative interventions.^2^, ^3^ The management of advanced tooth wear, tooth fractures, and developmental tooth defects may also necessitate restorative treatment.^6^ These restorative treatments protect the dentin‐pulp complex, prevent lesion progression, and restore cleanability, form, function, and esthetics.^2^, ^3^, ^6^, ^7^ The overarching goal is to conserve as much sound tooth structure as possible and maintain pulp vitality whenever feasible.^2^, ^3^, ^6^, ^8^, ^9^, ^10^, ^11^

Advancements in adhesive dentistry have broadened the application scope for both direct and indirect restorations, blurring the lines between the two approaches.^12^, ^13^, ^14^ Direct restorations have proven effective for posterior teeth with extensive defects, including cases necessitating cusp replacement.^15^, ^16^, ^17^, ^18^ Indirect restorations provide an alternative for the restorative management of such teeth, offering enhanced control over the restoration contour and occlusion, particularly in cases that require complex restorative rehabilitation.^12^

Inlays and onlays are partial coverage restorations for posterior teeth. Inlays restore tooth structure without extending over the cusps, whereas onlays provide coverage for one or more cusps. Overlays, a specific type of onlay, encompass all of the tooth's cusps. When direct restorations are not feasible, such as with wide interproximal gaps, inlays and onlays offer a minimally invasive alternative.^12^, ^19^ Compared with traditional crowns, partial coverage restorations conserve more tooth structure.^10^, ^20^, ^21^, ^22^, ^23^ The conservation of sound enamel and dentin aligns with the principles of minimally invasive dentistry, making inlays and onlays a preferred choice over crowns.^10^, ^12^, ^23^, ^24^, ^25^, ^26^

Various fabrication workflows, from laboratory techniques to chairside computer‐assisted design and computer‐assisted manufacturing (CAD‐CAM), enable the creation of indirect restorations using durable ceramics or other nonmetallic biomaterials.^27^, ^28^, ^29^, ^30^ Systematic reviews of clinical studies have shown that inlays and onlays obtain a satisfactory level of long‐term success, with survival rates ranging from 85% to 91% after a decade of follow‐up.^31^

Despite these advancements, a significant gap remains in the literature concerning the optimal use of ceramic and nonmetallic biomaterials for indirect restorations in posterior teeth.^11^ Additionally, the prevailing fee‐for‐service dental economic models have potential to foster an environment prone to overtreatment, leading to unnecessary restorative procedures that compromise oral health outcomes and waste resources.^32^ This study aims to address these issues by providing evidence‐based consensus recommendations derived from systematic reviews and a two‐phase e‐Delphi survey.^33^ These recommendations seek to fill the literature gap and promote judicious, effective treatment practices in restorative dentistry.

The primary objective of this study is to develop consensus recommendations for the use of ceramic and nonmetallic biomaterials in single indirect restorations for posterior teeth. This interdisciplinary Delphi study, conducted by the PROgress in Science and Education with Ceramics (PROSEC) North America panel, aims to integrate findings from systematic reviews with expert consensus to establish a framework that supports dental practitioners in making informed choices and delivering high‐quality restorative care for patients. The recommendations do not seek to update or supersede existing guidance for restorative patient care.^12^, ^34^, ^35^, ^36^

## 2 METHODS

### 2.1 Multidisciplinary panel

PROSEC is an interdisciplinary and international expert network dedicated to advancing science and continuous education focused on ceramic and alternative metal‐free restorations. PROSEC fosters professional collaborations spanning universities, dental practices, and laboratories. The PROSEC North America panel, which formulated the recommendations put forward in this report, comprised 16 panelists. The panel encompassed dental practitioners primarily specializing in preventive dentistry, cariology, restorative dentistry, and prosthodontics, along with dental researchers, technicians, and educators. The PROSEC North America Board selected panelists by consensus from among PROSEC North America members and associated experts. The selection criteria included expertise in dental biomaterials and restorative dentistry, along with involvement in dental research and/or education, ensuring a diverse and knowledgeable panel. Some panelists were affiliated with the same institution. No specific weighting or adjustments accounted for this during the consensus process.

### 2.2 Consensus process

The panel convened for three separate 1‐day consensus workshops in Philadelphia, PA, USA on June 30, 2023, and in Chicago, IL, USA on November 10, 2023, and February 22, 2024.

The initial workshop was dedicated to a comprehensive discussion and determination of the objective, scope, and strategies for the publication and dissemination of the consensus report. Furthermore, it involved a collaborative and thorough preliminary assessment of relevant clinical studies and systematic reviews, which informed the methodology to be adopted in the subsequent phases of developing the consensus recommendations.

### 2.3 Systematic literature review

After the initial workshop, a systematic literature review was conducted to gather insights from systematic reviews of clinical studies evaluating indirect restorations made from ceramic and nonmetallic biomaterials in posterior teeth.

### 2.4 Eligibility criteria

Systematic reviews with full study reports were selected based on the inclusion and exclusion criteria. Only systematic reviews of clinical studies were eligible for inclusion, because they provide a comprehensive synthesis of evidence from multiple studies, offering high‐level evidence that informs clinical practice. There were no constraints on publication date or language.

### 2.5 Inclusion criteria

- Systematic reviews of clinical studies
- Indirect restoration of posterior permanent teeth
- Restoration type: inlays, onlays, overlays, endocrowns, or single‐unit crowns
- Biomaterial: glass ceramics, oxide ceramics, polymer‐infiltrated ceramic network materials, indirect resin‐based composites, CAD‐CAM resin‐based composites

### 2.6 Exclusion criteria

- Narrative reviews
- Systematic reviews of in vitro, in silico, or animal studies
- Summaries of systematic reviews
- Meta‐analyses without systematic literature search
- Posters or abstract‐only articles
- Withdrawn reports
- Restoration of deciduous teeth
- Inlay‐retained or onlay‐retained fixed dental prostheses
- Restoration of dental implants

### 2.7 Search strategy

On October 28, 2023, electronic searches were conducted across five databases: Cochrane Library, Embase, OpenGrey through DANS EASY Archive, PubMed, and Scopus. Query strings, detailed in Table S1, were adapted to align with the controlled vocabulary and syntax rules of each database. Additionally, the reference lists of systematic reviews meeting the eligibility criteria were manually examined to enhance the search.

### 2.8 Selection process

Duplicates were manually removed by F. E. Subsequently, F. E. and J. C. independently assessed the titles and abstracts of the retrieved articles against the eligibility criteria, selecting those potentially relevant for the recommendation creation. Throughout this process, both author names and journal titles were unblinded. Full articles of these potentially relevant systematic reviews were acquired for the next phase. F.E. and J.C. independently assessed the retrieved articles against the eligibility criteria. Any disagreements about study eligibility were initially resolved through discussion between the two primary reviewers. Though the methodological framework included the option to consult a third reviewer for unresolved disagreements, this step was not required during the conduct of the study as all disagreements were successfully resolved through discussion. The reasons behind any exclusions were noted. Systematic reviews that had multiple associated publications were cross‐referenced and connected. All panelist received the full texts of the selected systematic reviews and pertinent consensus reports for thorough evaluation.^2^, ^3^, ^6^, ^34^, ^35^, ^36^

### 2.9 Development of draft recommendations

Taking into account the systematic reviews meeting the eligibility criteria and structured consensus reports, the panel formulated recommendations during the second workshop session. This process included a comprehensive incorporation of insights from all panelists. Each recommendation was subject to thorough discussion and iterative refinement to ensure clarity and consensus. Following deliberations during the second workshop, the steering group, represented by A. L. O., F. E., and J. C., wrote the initial draft of the report.

### 2.10 Delphi process

A confidential, two‐stage e‐Delphi survey was initiated using an online survey platform (EvaSys, Lüneburg, Germany).^33^ Each e‐Delphi survey round was structured to last a fortnight, with a reminder email sent to participants midway through the period to encourage responses.

The e‐Delphi survey enabled panelists to anonymously register their level of agreement for each recommendation on a scale of 1 (strongly disagree) to 10 (strongly agree).^2^, ^3^ Moreover, in the first e‐Delphi round, a dedicated section was incorporated for free‐text feedback, providing panelists an avenue to elucidate their rationale or suggest potential amendments.

Following the first e‐Delphi round, a second e‐Delphi round permitted anonymous voting along the same scale. Outcomes were categorized as agreement (scores 8–10), neutral (scores 4–7), or disagreement (scores 1–3). A recommendation was deemed accepted by the panel if it secured a vote above seven from at least 70% of the panelists.^2^, ^3^ The mean and standard deviation of agreement scores were also computed for each statement.

The decision to use an e‐Delphi survey was driven by the suitability of this approach for synthesizing insights beyond the confines of randomized controlled trials and the objective to systematically establish consensus to address uncertainties in restorative care for posterior teeth.

### 2.11 Publication and dissemination

This report, reviewed and unanimously endorsed by all panelists, presents the consensus recommendations. The strategy for publication and dissemination, established in the initial workshop, targeted open‐access publication in the *Journal of Esthetic and Restorative Dentistry*. This approach was chosen to reach the broad readership of the journal and to provide the wider dental community with unrestricted access to the report.

## 3 RESULTS

### 3.1 Systematic literature review

**FIGURE 1:**
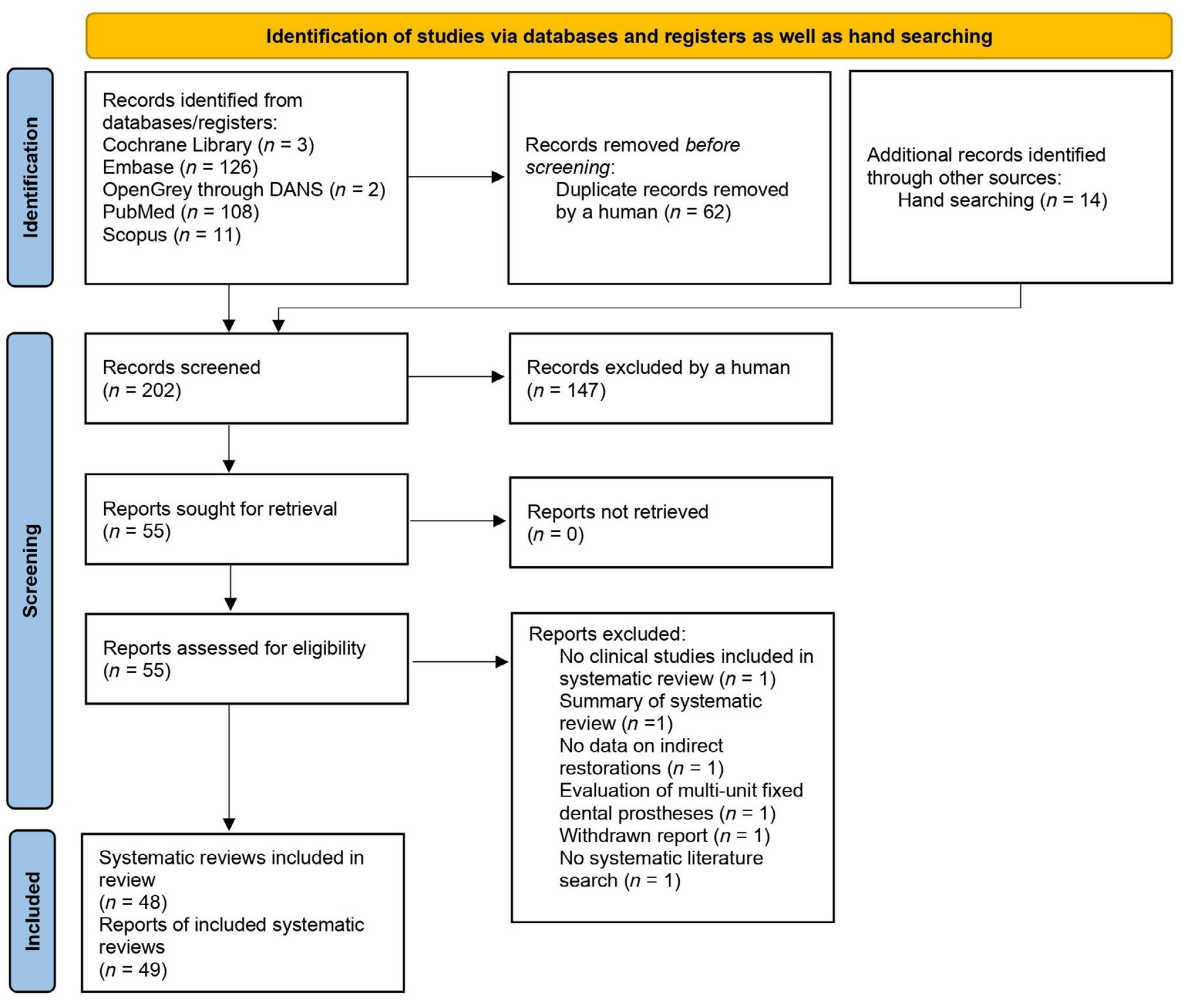
PRISMA 2020 flow diagram^84^ illustrating the record selection process used for the development of consensus recommendations.

Figure 1 illustrates the study selection process, which included 48 systematic reviews that corresponded to 49 published reports.^16^, ^31^, ^37^, ^38^, ^39^, ^40^, ^41^, ^42^, ^43^, ^44^, ^45^, ^46^, ^47^, ^48^, ^49^, ^50^, ^51^, ^52^, ^53^, ^54^, ^55^, ^56^, ^57^, ^58^, ^59^, ^60^, ^61^, ^62^, ^63^, ^64^, ^65^, ^66^, ^67^, ^68^, ^69^, ^70^, ^71^, ^72^, ^73^, ^74^, ^75^, ^76^, ^77^, ^78^, ^79^, ^80^, ^81^, ^82^, ^83^ Detailed information on these systematic reviews can be found in Table S2. Table S3 provides an overview of the records excluded following the full‐text assessment.

### 3.2 Key recommendations

Table 1 enumerates the five key recommendations established by PROSEC North America.

**TABLE 1:** Key recommendations regarding single indirect restorations made from ceramic and nonmetallic biomaterials for posterior teeth.

No.	Recommendation	Agreement^a^
1	***Comprehensive management strategy and customized treatment planning***	8.8 (2.3)
	Develop a comprehensive, individualized treatment plan that addresses the underlying pathology, such as dental caries or tooth wear. Ensure that the criteria for transitioning from noninvasive or microinvasive techniques to restorative interventions are appropriately chosen to prevent overtreatment. Prioritize strategies that create favorable conditions for a tooth's long‐term prognosis and the dentition's overall functionality.	
	Alongside restorative interventions, comprehensive treatment must also encompass effective noninvasive measures to optimize oral health outcomes. Case selection is crucial in determining which patients would benefit most from which type of intervention. When considering restorative treatment, both in its planning and execution, the emphasis should be on the overall condition and prognosis of the teeth rather than concentrating solely on the lifespan of individual restorations. The choice between direct restoration and indirect restoration should be based on a thorough assessment and a collaborative decision‐making process between dental practitioners and patients. To manage partially defective restorations, conservative methods should be the first choice. It is often advantageous to opt for restoration refurbishment or repair as primary intervention rather than resorting to complete restoration replacement. The success of restorative treatments hinges on a variety of factors, including individual patient characteristics, the specific conditions of the tooth in question, the skill and techniques of the dental practitioner, and the properties of the biomaterials employed. This necessitates a personalized treatment approach that thoughtfully considers these varied factors. Such an approach ensures that each patient receives care that is not only technically proficient but also tailored to their unique dental needs.	
2	***Minimizing tooth substance loss***	8.2 (3.0)
	Adopt defect‐oriented preparation and adhesive techniques to minimize the loss of sound tooth structure.	
	The indirect restoration preparation design should consider defect morphology, promote favorable biomechanical stress distribution, prevent areas of internal stress concentration, and factor in occlusal considerations. The preparation design must also account for patient‐specific risks such as bruxism, consider biomaterial specifications, ensure conditions conducive to optimal restoration placement, and incorporate esthetic considerations. Using direct restorative materials for undercut blocking and buildup allows for cavity design optimization, enabling minimally invasive tooth preparation. Bonded indirect restorations obviate the need for retention and resistance form preparation. Evaluating each tooth to determine the need for cuspal coverage is essential. In cases where cuspal coverage is deemed necessary, using bonded partial coverage restorations is advisable when clinical parameters allow.	
3	***Precision in clinical procedures***	8.9 (2.4)
	Execute procedures with meticulous care.	
	Ensuring effective tooth preparation design, impression procedures, contamination control, and bonding procedures is crucial for all ceramic and nonmetallic restorations. If necessary, careful gingival tissue retraction must be achieved. For adhesive procedures, contamination control through adequate isolation is imperative. When adjusting proximal contacts and the occlusion, scrupulous care is paramount to mitigate grinding‐induced surface and subsurface damage, especially in brittle biomaterials.	
4	***Biomaterial selection and use***	8.7 (2.4)
	When selecting and using restorative biomaterials, prioritize materials that excel in clinical performance and meet esthetic requirements.	
	In selecting biomaterials, it is essential to acknowledge variations in clinical performance and their esthetic properties. The choice of biomaterials should be based on the best available evidence and consider the dental practitioner's expertise, patient preferences, esthetic requirements, and, when relevant, compliance with insurance or regulatory stipulations. In every case, the balance of mechanical and esthetic properties needs careful assessment. Restorative biomaterials ought to be utilized as per manufacturer guidelines. For adhesive procedures, regardless of the adhesive type—self‐etch, etch‐and‐rinse, or universal—using phosphoric acid etching on enamel is considered beneficial. For bonding to dentin, a range of proven techniques is available to ensure reliable adhesion.	
5	***Follow‐up and maintenance***	8.9 (2.2)
	Educate patients about proper oral hygiene practices and emphasize the importance of regular follow‐ups and monitoring.	
	Educating patients on appropriate oral hygiene practices is essential. Tailoring regular monitoring and follow‐up to each patient's specific needs is critical for sustaining preventive measures and assessing the condition of restored teeth and the functional integrity of restorations. Periodic evaluations by dental practitioners are crucial to early identification of issues such as wear, defects, or other concerns, steering appropriate management. These evaluations should follow established criteria for assessing teeth with restorations. Patients should also receive maintenance care, including professional removal of biofilm, calculus, or stains as necessary.	

^a^ Mean agreement scores with standard deviations in parentheses.

## 4 DISCUSSION

### 4.1 Significance of the PROSEC North America recommendations

While many of these recommendations align with established principles in restorative dentistry,^12^, ^34^, ^35^, ^36^ they have been synthesized to reflect the most current evidence and expert consensus, ensuring their relevance and applicability to contemporary clinical practice. Given the susceptibility of dental care to overdiagnosis and overtreatment, which contravenes the principles of evidence‐based dentistry, the role of evidence‐based decision‐making in restorative dentistry is particularly critical.^32^ The choice of treatment, including the prudent decision to refrain from invasive interventions, is vital for optimal oral health outcomes and efficient resource allocation.^32^ Therefore, these PROSEC North America recommendations emphasize the importance of thorough treatment planning, minimally invasive approaches, and practices designed to deliver effective care while avoiding overtreatment.

### 4.2 Comprehensive management strategy and customized treatment planning

Restorative interventions remain important in clinical patient care. Still, they should always be part of a broader strategy that includes noninvasive or microinvasive approaches to control the underlying oral disease, such as caries or tooth wear.^2^, ^3^ To achieve successful treatment outcomes, it is essential to identify, understand, and manage individual patient risks. The decision to proceed with restorative intervention is guided by factors such as lesion activity, cavitation status, and the tooth's cleanability.^2^ Patients must be capable and willing to perform effective biofilm control before initiating definitive restorative procedures.

Preventive measures are paramount in managing tooth wear and should precede any restorative interventions. These measures, aimed at arresting or decelerating tooth wear progression, form the foundation of treatment, enhancing the efficacy and durability of subsequent restorative treatments.^6^, ^41^

In the treatment planning phase, the emphasis should be on the restored tooth's long‐term health and functionality and patient‐centered outcomes rather than on restoration survival and success rates.^12^ Selecting between direct and indirect restorations requires a personalized approach. While direct restorations are generally less invasive and more cost‐effective, indirect restorations might be more suitable in complex cases where achieving optimal form, esthetics, or managing occlusal dynamics with direct restorations is challenging.^12^ Dental practitioners need to consider their expertise, the defect's characteristics, the complexity of the case, esthetic goals, and patient expectations.

Refurbishing or repairing partially defective restorations often presents advantages over opting for complete replacements.^12^, ^35^ A replacement restoration typically involves removal of sound tooth structure.^3^, ^36^ Refurbishing or repairing the existing restoration can be a more conservative approach, so dental practitioners need to view refurbishment and repair as viable options.^3^, ^12^ Guidelines for clinical and radiographic evaluations provide direction on various management options, including monitoring, refurbishing, repairing, and replacing restorations.^36^ However, it is important to note that these guidelines serve as flexible frameworks to assist in decision‐making, allowing for treatments to be tailored to the specific needs and preferences of both the dental practitioner and the patient, rather than being prescriptive mandates.^36^

### 4.3 Minimizing tooth substance loss

Conserving healthy tooth structure is a basic tenet of minimally invasive dentistry. Adhesive techniques provide microretention for bonded restorations, eliminating the need for the macroretention achieved through traditional retention and resistance form preparation.^23^ Adhesive techniques favor defect‐oriented preparation designs, which conserve sound enamel and dentin without reducing fracture resistance or increasing stress concentration.^25^, ^26^ In addition, such designs improve the conditions for bonding and reduce the potential for pulpal damage.^24^

Given the reliable long‐term performance of partial coverage restorations, the use of crowns is now considered highly specific, restricted mainly to situations where an existing crown needs replacement or when crowns are required as parts of fixed dental prostheses.^12^

Utilizing adhesive buildup techniques before the preparation for an indirect restoration is recommended. This approach enhances the tooth's structural integrity and facilitates a minimally invasive preparation design. Without such buildup, eliminating undercuts might require the removal of significant amounts of healthy tooth structure, which contradicts the principles of minimal invasiveness. If the dental practitioner's preference is to perform the buildup after tooth preparation, scrupulous care must be taken during preparation to visualize areas of undercuts and plan to adhesively block these areas as opposed to extending the preparation for the path of indirect restoration placement. When performing the adhesive buildup, the technique should attempt to maximize the bond to dentin by controlling C‐factor and shrinkage stress during polymerization. Performing an immediate dentin sealing and resin coating technique may also be considered.^24^

The preparation design, smooth and without abrupt transitions, should promote favorable distribution of masticatory forces.^24^ The design should help dissipate forces from the periphery to the tooth's center and minimize tensile stress within the restoration and at the adhesive interface.^24^ The preparation margins, ideally located in enamel, must be clearly delineated to ensure the optimal fit of the restoration, both internally and at the margins.^24^ Cutting enamel prisms obliquely can enhance bonding.^24^ Furthermore, the minimal thickness specifications of each biomaterial dictate the requisite reduction during preparation.

Teeth with root canal treatment often present with significant loss of tooth structure, including the access cavity, making it imperative to consider cuspal coverage.^13^ Though partial coverage restorations tend to have a higher failure rate in endodontically treated teeth compared with those that are vital, it is noteworthy that most of these failures are repairable.^22^ This suggests that partial coverage restorations can effectively preserve endodontically treated teeth while also conserving healthy tooth structure.^13^

Managing posterior teeth with cracks is often complex. It is essential to assess the location and extent of cracks along with signs and symptoms of cracked tooth syndrome—or note their absence. For teeth affected by cracked tooth syndrome, it is recommended to use restorations that cover all cusps.^7^, ^9^ This approach aids in the favorable distribution of masticatory forces and lowers the risk of tooth loss.^7^

Restoring teeth with subgingival defects poses a set of challenges. Deep margin elevation can be a less invasive alternative to surgical crown lengthening in some cases, facilitating impression taking and the delivery of indirect restorations.^20^, ^21^ However, it is important to acknowledge that the evidence supporting this restorative approach mainly stems from laboratory studies and a limited number of clinical trials, indicating the need for cautious application in practice.^11^

### 4.4 Precision in clinical procedures

Restorative procedures demand precise execution. The dental practitioner's experience and skills influence the success of both direct and indirect restorations, often surpassing the impact of the materials employed.^17^, ^19^ For favorable long‐term outcomes, it is imperative to conduct every phase of the treatment with meticulous attention and to allot sufficient time for each appointment, ensuring the careful completion of each step.

For the success of adhesive procedures, stringent contamination control is imperative. Isolation with rubber dam represents the most dependable means of ensuring a clean and dry working field. Beyond contamination control, rubber dam isolation offers multiple advantages, including protecting soft tissues, minimizing aerosol and droplet generation, preventing ingestion and aspiration incidents, and retracting gingival tissue. A systematic review of randomized controlled trials indicates that there is low‐certainty evidence suggesting that the use of a rubber dam in adhesive restorative procedures might lead to a lower rate of restoration failures compared with the use of cotton rolls after a 6‐month observation period.^85^ Additionally, a recent in situ study found that rubber dam isolation enhances enamel bond strength, irrespective of the adhesive employed.^86^ Considering its manifold practical benefits and the low‐certainty evidence supporting its positive impact on treatment outcomes, the use of rubber dam isolation is recommended during adhesive procedures, encompassing both buildup and bonding of indirect restorations. As alternatives to rubber dam, other dental isolation systems with mouthpieces providing retraction and continuous evacuation of fluids may be considered.^12^, ^87^

When adjusting interproximal contact points or occluding surfaces, scrupulous care is essential to minimize the risk of restoration damage from grinding, particularly in brittle biomaterials such a glass ceramics.^30^ Laboratory research indicates that polymer‐infiltrated ceramic network materials and CAD‐CAM resin‐based composites are less susceptible to such damage.^30^ Nonetheless, irrespective of the biomaterial, one should apply minimal pressure during grinding and opt for either red ring “fine” (45–50 μm grit size) or yellow ring “superfine” (15–30 μm grit size) diamond burs.^30^ Following up with biomaterial‐specific high‐gloss polishing is necessary to enhance the result. Polishing is also beneficial to reduce tooth wear on the antagonist.^73^

### 4.5 Biomaterial selection and use

Today, a wide array of biomaterials exists for fabricating indirect restorations for posterior teeth. These biomaterials differ in their composition, their physical and mechanical properties, esthetic qualities, machinability, cost, and the intricacy of the steps involved in restoration fabrication.^27^ Notably, based on their popularity and the time of market introduction, there are marked variations in the volume of laboratory and clinical studies examining a particular biomaterial and the duration of follow‐up. Consequently, when selecting a biomaterial, dental practitioners are advised to weigh the evidence on the performance record of a specific biomaterial, individual case necessities, their clinical expertise, and patient preferences.

There is limited comparative data on the long‐term performance of different biomaterials for indirect restorations in posterior teeth. Nonetheless, some evidence indicates that ceramic inlays and onlays tend to surpass counterparts made from resin‐based composite in performance.^10^, ^72^ Among ceramic biomaterials, lithium disilicate is distinguished by its particularly robust performance in posterior teeth.^10^, ^28^, ^29^

Layered and veneered restorations are more prone to technical complications such as chipping.^27^, ^67^ As a result, there has been a shift toward monolithic restorations or restorations that are minimally veneered, avoiding veneering ceramic in areas with functional contacts.^27^ Given the satisfactory esthetic outcomes achievable with monolithic restorations in posterior teeth, opting for these solutions is often a judicious strategy to minimize the likelihood of complications.

Gold and metal‐ceramic restorations obtain favorable long‐term results, with some systematic reviews noting reduced annual failures rates compared with all‐ceramic restorations.^55^ This suggests that these biomaterials remain a valid and reliable option for specific clinical scenarios.

Following manufacturer guidelines is essential for maximizing the durability of indirect restorations and ensuring compliance with regulatory standards. Adhering to these detailed instructions can prevent potential problems affecting the structural integrity and functionality of restorations.

When choosing adhesives, dental practitioners should consider their performance record in both laboratory and clinical studies. Though all adhesive strategies, including self‐etch, etch‐and‐rinse, and selective enamel etching, are valid options, phosphoric acid etching on enamel is recognized as a beneficial conditioning step, irrespective of the adhesive type used.^18^

Immediate dentin sealing entails applying an adhesive, optionally combined with a layer of flowable resin‐based composite, onto exposed dentin immediately following tooth preparation and prior to impression taking.^88^ While laboratory research indicates that immediate dentin sealing enhances the bond strength of indirect restorations, there is a paucity of clinical evidence supporting its benefits.^14^, ^89^ This suggests that immediate dentin sealing may be considered, but it is not a requisite step for the success of indirect restorations.

### 4.6 Follow‐up and maintenance

Follow‐up and maintenance care can enhance the longevity of indirect restorations.^24^ The frequency of follow‐up visits should be tailored to each patient's unique needs, risk factors, and preferences.

During these visits, it is recommended to follow established guidelines, such as the revised FDI criteria, for evaluating teeth with restorations to ensure precise assessment and appropriate management of defects while preventing unnecessary treatments.^36^ For instance, it is important to assess occlusion and articulation, making adjustments as needed to address premature contacts, hyperocclusion, or balancing interferences through restoration refurbishment.^24^, ^36^ Any rough surfaces should be repolished to minimize biofilm accumulation and mitigate the risk of fractures.^24^

The role of self‐performed oral hygiene in maintaining periodontal health and preventing secondary caries cannot be overstated. Regular follow‐up appointments are vital for monitoring periodontal status, removing microbial deposits, and encouraging patients to sustain or enhance their oral hygiene practices.^11^

Parafunctional behaviors, such as sleep bruxism, may pose risks to the integrity of restorations.^42^ Though a systematic review found no correlation between sleep bruxism and the failure of ceramic restorations in posterior teeth,^90^ it remains prudent to evaluate the potential benefits of an occlusal splint on an individual basis. For patients with existing occlusal splints, ensuring proper adjustment or creating a new splint post‐restoration is advisable to maintain optimal fit and protection.

### 4.7 Limitations of these recommendations

It is important to consider the limitations of the recommendations put forward in this article.

The absence of a structured assessment regarding the certainty of evidence supporting our recommendations represents a salient limitation. While tools such as the GRADE system provide systematic evaluations of evidence quality,^91^ integrating such in‐depth appraisals was outside the scope of this study. Readers must recognize that the evidence underpinning these consensus recommendations typically ranges from low to very low certainty. Given the often nuanced balance between benefits and risks in restorative patient care, coupled with the variability in patient values and preferences, and the disparate cost structures and renumeration systems across dental healthcare systems, these recommendations carry a weak strength. This underscores the importance of individualized treatment choices that align with the dental practitioner's expertise, the context of practice, and the individual patient's unique values and preferences.

While this study utilized a systematic literature search to gather evidence from systematic reviews, it is essential to distinguish this approach from conducting a full umbrella review. The systematic literature search compiled existing systematic reviews to inform the consensus process, but it did not involve the rigorous, standardized methodology and comprehensive risk of bias assessment that characterize an umbrella review. This methodological choice was made to efficiently consolidate high‐level evidence and expert opinions within a practical timeframe. However, the omission of a formal risk of bias assessment for each included systematic review is a notable limitation. The PROSEC North America panel acknowledges that, without a structured assessment tool like AMSTAR, there remains an inherent risk of overlooking subtle biases within the included reviews.^92^ This limitation ought to be considered when interpreting these recommendations.

The panel utilized a combination of an open‐ended approach and an e‐Delphi survey to foster a systematic and comprehensive exploration. The panel was assembled from a diverse group of dental professionals. However, the absence of representation from patient groups is a limitation of our approach. We strongly encourage input from patient groups to ensure that their perspectives are incorporated in future versions of the recommendations. We also invite dental professionals and other stakeholders to provide constructive feedback and participate in the ongoing development of these recommendations.

PROSEC promotes science and education centered on ceramic and alternative metal‐free restorations. Readers need to know that this article focuses on these types of biomaterials rather than the full spectrum of options for restoring posterior teeth with extensive defects. Furthermore, considering PROSEC's goals as stated in its mission, there is an acknowledged risk of bias in the panel's recommendations, potentially leaning the assessment of evidence toward favoring ceramic and metal‐free alternatives.

As research insights in dentistry evolve, it is important to regularly review and update recommendations for restorative patient care. PROSEC North America is dedicated to reviewing these recommendations every five years or sooner if significant new evidence becomes available.

## 5 CONCLUSIONS

The PROSEC North America consensus recommendations are designed to improve patient outcomes, reduce overtreatment, and ensure prudent resource allocation in restorative dentistry. From these recommendations, the following key takeaways emerge:

- *Integrative approach*: Restorative interventions must be part of a comprehensive strategy that includes noninvasive measures to effectively manage dental caries and tooth wear.
- *Personalized treatment*: The choice between direct and indirect restorations should be individualized, taking into account the complexity of the case, esthetic demands, and patient preferences.
- *Preference for minimally invasive techniques*: Inlays and onlays are preferred over crowns whenever possible to conserve more tooth structure, in line with minimally invasive principles.
- *Evidence‐based biomaterial selection*: The selection and use of restorative biomaterials should be guided by clinical performance, esthetic considerations, and strict adherence to manufacturer guidelines.
- *Precision in clinical procedures*: Meticulous execution in tooth preparation, impression taking, contamination control, and luting is essential for the success of ceramic and nonmetallic restorations.
- *Regular follow‐up and maintenance*: Tailored follow‐up and maintenance are essential for the longevity of teeth with indirect restorations. Adherence to established guidelines for evaluating restorations is crucial to avoid overtreatment and ensure optimal patient outcomes.

## CONFLICT OF INTEREST STATEMENT

Victor E. Castro declares payment or honoraria for lectures, presentations, speakers bureaus, manuscript writing, or educational events from Anaxdend and VITA. Jakob Brief serves as the CEO of PROSEC gGmbH and holds the position of Head of Scientific & Clinical Management at VITA Zahnfabrik, both organizations are based in Bad Säckingen, Germany. Miguel A. Oritz declares payment or honoraria for lectures, presentations, speakers bureaus, manuscript writing, or educational events from Kuraray Noritake USA and VITA North America. Miguel A. Oritz also declares support for attending meetings and/or travel from Kuraray Noritake USA and VITA North America. Peter Pizzi declares payment or honoraria for lectures, presentations, speakers bureaus, manuscript writing, or educational events from Amman Girrbach and VITA. Michael J. Tholey serves as the Head of Education and Training at VITA Zahnfabrik in Bad Säckingen, Germany. Awab Abdulmajeed, Wael Att, Markus B. Blatz, Julián Conejo, Lyndon F. Cooper, Florin Eggmann, Amelia L. Orta, and Rade D. Paravina declare no competing interests.

## Supporting information


**Data S1.** Supporting Information.

## Data Availability

All data supporting the findings of this article are comprehensively contained within the main text and the accompanying supporting information.
