# Supplementary material for: Interdisciplinary Delphi study by PROSEC North America: Recommendations on single indirect restorations made from ceramic and nonmetallic biomaterials for posterior teeth
Source: J Esthet Restor Dent. 2024 Aug 2;37(3):809–20. doi: 10.1111/jerd.13289 (PMC12076105; doi:10.1111/jerd.13289)
Supplement: Supplementary file 1 — Data S1. Supporting Information. [file JERD-37-809-s001.pdf]

PROSEC North America consensus recommendations: Single indirect restorations made from ceramic and non-metallic biomaterials for posterior teeth

SUPPLEMENTARY MATERIAL

Supplementary Table S1 Details on the electronic searches performed on October 28, 2023

**Database**	**Query string**	**Records identified**
Cochrane Library	(posterior tooth OR posterior teeth OR molar OR premolar OR bicuspid) AND (inlay OR onlay OR overlay OR partial coverage restoration OR crown OR single tooth restoration) AND (ceramics OR all-ceramic OR porcelain OR zirconia OR zirconium dioxide OR silicate ceramic OR feldspar ceramic OR felspathic ceramic OR glass ceramic OR oxide ceramic OR lithium silicate OR zirconia-reinforced lithium silicate OR leucite-reinforced lithium silicate OR lithium disilicate OR alumina ceramic OR alumina-reinforced ceramic OR alumina-based ceramic OR hybrid ceramic OR polymer-infiltrated ceramic network OR polymer-infiltrated ceramic OR organically modified ceramics OR Enamic OR CAD-CAM resin-based composite OR resin-nano ceramic OR nano-ceramic OR nano-ceramic resin composite) AND (systematic review OR review OR meta-analysis) in Title Abstract Keyword - (Word variations have been searched)	3
Embase	(posterior tooth OR posterior teeth OR molar OR premolar OR bicuspid) AND (inlay OR onlay OR overlay OR partial coverage restoration OR crown OR single tooth restoration) AND (ceramics OR all-ceramic OR porcelain OR zirconia OR zirconium dioxide OR silicate ceramic OR feldspar ceramic OR felspathic ceramic OR glass ceramic OR oxide ceramic OR lithium silicate OR zirconia-reinforced lithium silicate OR leucite-reinforced lithium silicate OR lithium disilicate OR alumina ceramic OR alumina-reinforced ceramic OR alumina-based ceramic OR hybrid ceramic OR polymer-infiltrated ceramic network OR polymer-infiltrated ceramic OR organically modified ceramics OR Enamic OR CAD-CAM resin-based composite OR resin-nano ceramic OR nano-ceramic OR nano-ceramic resin composite) AND (systematic review OR review OR meta-analysis)	126
OpenGrey through DANS EASY Archive	(posterior tooth OR posterior teeth OR molar OR premolar OR bicuspid) AND (inlay OR onlay OR overlay OR partial coverage restoration OR crown OR single tooth restoration) AND (ceramics OR all-ceramic OR porcelain OR zirconia OR zirconium dioxide OR silicate ceramic OR feldspar ceramic OR felspathic ceramic OR glass ceramic OR oxide ceramic OR lithium silicate OR zirconia-reinforced lithium silicate OR leucite-reinforced lithium silicate OR lithium disilicate OR alumina ceramic OR alumina-reinforced ceramic OR alumina-based ceramic OR hybrid ceramic OR polymer-infiltrated ceramic network OR polymer-infiltrated ceramic OR organically modified ceramics OR Enamic OR CAD-CAM resin-based composite OR resin-nano ceramic OR nano-ceramic OR nano-ceramic resin composite) AND (systematic review OR review OR meta-analysis)	2
PubMed	(posterior tooth OR posterior teeth OR molar OR premolar OR bicuspid) AND (inlay OR onlay OR overlay OR partial coverage restoration OR crown OR single tooth restoration) AND (ceramics OR all-ceramic OR porcelain OR zirconia OR zirconium dioxide OR silicate ceramic OR feldspar ceramic OR felspathic ceramic OR glass ceramic OR oxide ceramic OR lithium silicate OR zirconia-reinforced lithium silicate OR leucite-reinforced lithium silicate OR lithium disilicate OR alumina ceramic OR alumina-reinforced ceramic OR alumina-based ceramic OR hybrid ceramic OR polymer-infiltrated ceramic network OR polymer-infiltrated ceramic OR organically modified ceramics OR Enamic OR CAD-CAM	108

	resin-based composite OR resin-nano ceramic OR nano-ceramic OR nano-ceramic resin composite) NOT ("Dental Implants"[Mesh]) NOT ("In Vitro Techniques"[Mesh]) NOT ("Animals"[Mesh] NOT "Humans"[Mesh]) Filters: Meta-Analysis, Review, Systematic Review	
Scopus	(posterior tooth OR posterior teeth OR molar OR premolar OR bicuspid) AND (inlay OR onlay OR overlay OR partial coverage restoration OR crown OR single tooth restoration) AND (ceramics OR all-ceramic OR porcelain OR zirconia OR zirconium dioxide OR silicate ceramic OR feldspar ceramic OR felspathic ceramic OR glass ceramic OR oxide ceramic OR lithium silicate OR zirconia-reinforced lithium silicate OR leucite-reinforced lithium silicate OR lithium disilicate OR alumina ceramic OR alumina-reinforced ceramic OR alumina-based ceramic OR hybrid ceramic OR polymer-infiltrated ceramic network OR polymer-infiltrated ceramic OR organically modified ceramics OR Enamic OR CAD-CAM resin-based composite OR resin-nano ceramic OR nano-ceramic OR nano-ceramic resin composite) AND (systematic review OR review OR meta-analysis)	11

Supplementary Table S2 Overview of the records that were excluded after the full text assessment according to the eligibility criteria

Record	Reason for exclusion
Gevert MV, Soares R, Wambier LM, et al. How is the quality of the available evidence on molar-incisor hypomineralization treatment? An overview of systematic reviews. *Clin Oral Investig.* 2022;26(10):5989–6002. doi: 10.1007/s00784-022-04612-9.	No clinical studies included
Holme W. Gold versus ceramic - which will last longer for posterior indirect restorations? *Evid Based Dent.* 2022;23(4):166–167. doi: 10.1038/s41432-022-0837-7.	Summary of systematic review
Dashash M, Yeung CA, Jamous I, et al. Interventions for the restorative care of amelogenesis imperfecta in children and adolescents. *Cochrane Database Syst Rev.* 2013;2013(6. doi: 10.1002/14651858.CD007157.pub2.	No data on indirect restorations
Raigrodski AJ, Hillstead MB, Meng GK, et al. Survival and complications of zirconia-based fixed dental prostheses: A systematic review. *J Prosthet Dent.* 2012;107(3):170–177. doi: 10.1016/S0022-3913(12)60051-1.	Evaluation of multi-unit fixed dental prostheses
Hayashi M, Yeung CA. Ceramic inlays for restoring posterior teeth. *Cochrane Database Syst Rev.* 2003(1):CD003450. doi: 10.1002/14651858.CD003450.	Withdrawn report
Hickel R, Manhart J. Longevity of restorations in posterior teeth and reasons for failure. *J Adhes Dent.* 2001;3(1):45–64.	No systematic literature search

Supplementary Table S3 Overview of systematic reviews utilized for formulating consensus recommendations

**First author (year of publication)**	**Number of clinical studies included in the systematic review**	**Main findings**
Bresser et al. (2023)[1]	12	The findings from the systematic review and meta-analysis indicate that intracoronal restorations made from lithium disilicate and those made from indirect resin-based composites do not show any significant difference in terms of performance. There is medium-quality evidence suggesting that these biomaterials have equivalent survival rates over a short observation period. Additionally, the systematic review presented low-quality evidence that there is no statistically significant difference in survival rates between leucite restorations and indirect resin-based composite restorations. Conversely, intracoronal gold restorations demonstrated significantly better survival rates, establishing them as a more favorable choice compared with intracoronal indirect resin-based composite restorations.
Lopes-Fatturi et al. (2022)[2]	12	In first permanent molars with molars hypomineralization, the success rate of direct restorations varied between 86.3% and 100%, while indirect restorations had a success range from 91.3% to 100%.
Fathy et al. (2022)*[3]	7	The success rate of partial coverage restorations made from CAD-CAM resin-based composite ranged from 85.7 to 100% whereas the success rate reported for ceramic partial coverage restorations ranged from 93.3 to 100%. Fractures and debondings were found to be the most common cause of restorations failure.
Ferrari et al. (2022)[4]	1	The included randomized controlled clinical trial with a 3-year follow-up assessed posterior partial crowns made from lithium disilicate with or without posts. The study found no significant differences between teeth in which posts were luted and teeth restored without posts. The systematic review emphasized the need for further research regarding the design of all-ceramic partial crowns and the type of biomaterial to provide relevant data for the treatment of root filled posterior teeth.
Hardan et al. (2022)[5]	16	Clinical studies yield inconclusive results about the comparative clinical effectiveness of direct versus indirect restorations in treating tooth wear. The systematic review highlights the importance of personalized treatment planning and the necessity for further research to determine the most effective treatment protocols.
Leitão et al. (2022)[6]	9	The systematic review encompassed 594 participants and 1,657 monolithic single-unit zirconia crowns, with an average exposure time of 1.1 years and a follow-up ranging from 0.3 to 2.1 years. Survival rates across the studies varied from 91% to 100%. An exception was noted in a study involving patients with bruxism, which reported a lower survival rate of 31.60%. Marginal integrity generally showed high success rates throughout the observation periods. Linear regression analysis indicated that there was no statistically significant relationship between the survival rate and the type of luting protocol.
McGrath et al. (2022)[7]	30	The survival outcomes of the included studies ranged from 73.1% to 100%, with most studies reporting survival rates over 90%. The median observation time across studies

		was 5.1 years. The two studies containing direct resin-based composite placed as onlays studied a total of 155 restorations and found only seven failures, giving a combined 95.5% survival over five years. In the 28 studies containing 2,663 indirect onlays, the median restoration survival was 92.5% with a median observation of 5.1 years. The most frequent failure mode was restoration fracture. The most frequent deterioration criteria were loss of marginal integrity and discoloration.
Tennert et al. (2022)[8]	4	Evidence of low certainty suggests that ceramic restorations in posterior teeth have higher annual failure rates, between 2.1% and 5.6%, compared with gold restorations, which range from 0% to 2.1%. A meta-analysis revealed that conventionally cemented gold restorations demonstrated a significantly lower failure rate than adhesively-luted ceramic restorations, with a risk ratio of 0.31.
Aziz et al. (2020)[9]	6	The survival rates for lithium disilicate crowns fabricated with CAD-CAM technology were high in the short to medium term, with figures ranging between 93.3% and 100%. Biological complications, such as secondary caries and endodontic complications, occurred more frequently than technical complications associated with the crowns themselves. The frequency of complications showed no significant difference between premolars and molars.
Al-Dabbagh (2021)*[10]	3	The meta-analysis of the clinical studies showed an estimated overall 5-year survival rate of 91.4% for endocrowns and 98.3% for conventional crowns. The estimated overall 5-year success rates were 77.7% for endocrowns and 94% for conventional crowns. There were no significant differences in overall survival or success estimates between the assessed restorations.
Dioguardi et al. (2021)*[11]	8	The meta-analysis of the primary and secondary outcomes demonstrated that hazard ratios and survival rates seemed more favorable for indirect partial adhesive restorations on vital teeth than for those on endodontically treated teeth. Within the limits of this systematic review, these findings suggest that the risk of failure of indirect partial adhesive restorations on endodontically treated teeth is higher than on vital teeth.
Zou et al. (2021)[12]	5	Multi-surface inlays and onlays demonstrated comparable performance to that of one-surface inlays, with no evidence of inferiority.
Al-Haj Husain et al. (2020)[13]	28	All success rates decreased after 36 or more months compared to 24 months. The technical success rate of full crowns was statistically significantly higher than that of partial crowns.
Biagioni et al. (2020)*[14]	3	There is a paucity of evidence regarding full-mouth rehabilitation using adhesive partial crowns and the influence of occlusion in this context.
Govare et al. (2020)[15]	8	Survival rates for molar endocrowns exceeded 90% across a span of 6 months to 10 years and were found to be similar to those of traditional crowns. Conversely, premolar endocrowns had lower survival rates, varying from 68% to 75% at 55 months to 10 years, compared with 94% and 95% for traditional crowns on premolars. Loss of retention was the primary reasons for endocrown failure.
Komine et al. (2020)[16]	10	Indirect restorations made from a polymer-infiltrated ceramic network material and CAD-CAM resin-based composites showed a favorable short-term survival rate. Restoration fractures and debonding failure were the most

		frequently observed technical complications. Long-term data on clinical performance is lacking.
Papia et al. (2020)[17]	6	No conclusive evidence was found regarding the effect of preparation design, biomaterial, and luting protocol on the success and survival of endocrowns. The predominant causes of failure were loss of retention and fractures. Endocrowns in molars exhibited higher survival rates compared to premolars.
Solá-Ruíz et al. (2020)[18]	8	Natural teeth opposing monolithic zirconia crowns experience significant wear over time, which surpasses the wear observed on the crowns themselves. Factors affecting this wear include the surface treatment of the zirconia crowns, with greater wear caused by glazed surfaces compared with polished ones. Additionally, the wear is more pronounced in molars compared with premolars, varies by gender with less frequency in women than in men, and is influenced by the presence of parafunctional habits in patients.
Thomas et al. (2020)*[19]	8	Reported success rates of endocrown restorations in molars varied from 72.7% to 99.6% and in premolars ranged from 68.8% to 100%, with a follow-up range of 3-19 years. The pooled odds ratio and 95% confidence intervals for failure rates in molars compared with premolars in four studies selected for meta-analysis were 1.096 (95% confidence interval: 0.280, 4.292). These findings showed similar success rates and no difference in the rate of endocrown failures between molars and premolars, thus suggesting that premolars may be considered suitable candidates for endocrowns.
Vetromilla et al. (2020)[20]	43	Gold crowns (annual failure rate of 0.29%) and metal-ceramic crowns (annual failure rate of 0.52%) performed better for indirect restorations than metal-free restorations.
Gou et al. (2019)[21]	5	This systematic review indicated that the antagonist enamel wear caused by tooth-supported monolithic zirconia crowns in posterior teeth was comparable to, or greater than, that caused by natural teeth but less than that of metal-ceramic crowns.
Rodrigues et al. (2019)*[22]	14	The results of the meta-analysis indicate that tooth-supported ceramic restorations produced using CAD-CAM tend to have a shorter longevity compared with crowns fabricated through conventional methods.
Sampaio et al. (2019)*[23]	13	The estimated cumulative survival rate for ceramic and indirect composite inlays, onlays, and overlays made using CAD-CAM was 97% after 5 years and 89% after 10 years; for pressable was 95% after 5 years, and for stratified was 88% after 5 years and 93% after 10 years. Regardless of the manufacturing method, vitreous ceramic inlays, onlays, and overlays showed high survival, providing evidence that these restorations are a safe treatment.
Abduo et al. (2018)[24]	21	Medium-term studies (2-5 years) reported a survival rate for onlays between 91% and 100%, while long-term studies (over 5 years) showed a survival rate ranging from 71% to 98.5%. Fracture was identified as the most frequent cause of failure, followed by debonding and secondary caries. Onlay durability can be improved by designing preparations that provide at least 2 mm of occlusal ceramic thickness and include additional retentive elements. The likelihood of ceramic failure increases for restorations on root filled teeth, teeth located further back in the mouth, or teeth belonging to patients with parafunctional habits. The choice of biomaterial materials and fabrication method, as

		well as the adhesive bonding system, appeared to have no significant effect on the longevity of onlays.
Alves de Carvalho et al. (2018)[25]	29	After a mean follow-up duration of 7.0 years, endocrowns and crowns showed a significantly higher failure rate than inlays and onlays. After follow-up periods of at least three years, the overall survival rate of single-tooth ceramic restorations fabricated with CAD-CAM was similar to that of conventionally fabricated restorations.
Angeletaki et al. (2016)*[26]		The meta-analysis revealed that after 5 and 11 years, there was no statistically significant difference in failure risk between direct restorations and indirect inlays in posterior teeth. Marginal discoloration was the only parameter that was recorded more frequently for indirect inlays compared with direct restorations after 11 years.
Da Veiga et al. (2016)*[27]	9	The overall risk difference in longevity between direct and indirect resin-based composite restorations in permanent posterior teeth at five-year follow-up was 1.494 [0.893–2.500], and regardless of the type of tooth restored, that of molar and premolars was 0.716 [0.177–2.888] at three-year follow-up. The longevity of direct and indirect resin-based composite restorations was found to be similar, irrespective of the material used or whether the restored teeth were molars or premolars.
Morimoto et al. (2016)[28]	14	The meta-regression revealed that the survival rate of ceramic inlays and onlays was not significantly influenced by the type of ceramic material (feldspathic porcelain vs. glass-ceramic), the nature of the study design (retrospective vs. prospective), the length of follow-up (5 vs. 10 years), or the setting of the study (university vs. private clinic). Survival rates for glass-ceramics and feldspathic porcelain restorations were between 92% and 95% at 5 years, based on a sample of 5,811 restorations, and 91% at 10 years, with a sample size of 2,154 restorations. The most common reasons for failure were fractures/chipping at 4%, followed by endodontic complications at 3%, secondary caries at 1%, and debonding at 1%. There was no significant association between the incidences of failure associated with tooth type (premolars vs. molars). The chance of failure was 80% less (odds ratio 0.2) in vital teeth compared with endodontically treated teeth, implying that tooth vitality is a significant factor for restoration survival.
Kassardjian et al. (2016)[29]	14	The aggregated data encompassed 1,112 anterior all-ceramic crowns with 73 failures (6.5%) and 1,821 posterior all-ceramic crowns with 166 failures (9.1%), monitored over a period ranging from 3 years to 18.6 years. A relative risk meta-analysis of the included studies indicated that anterior all-ceramic crowns had a 50% lower likelihood of failure compared with posterior all-ceramic crowns
Sedrez-Porto et al. (2016)*[30]	3	The three clinical studies included in the analysis reported a success rate for endocrowns ranging between 94% and 100%.
Tsirogiannis et al. (2016)*[31]	4	Restorations created using digital impressions showed a mean marginal discrepancy of 56.1 micrometers, which was smaller than the 79.2 micrometers observed in restorations from conventional impressions. Meta-regression analysis, accounting for preparation design, found that digital impressions had, on average, 27.2 micrometers less marginal discrepancy than conventional methods, although this difference was not statistically significant.

Mangani et al (2015)[32]	32	Indirect ceramic restorations exhibited a success rate of 96.3%, which was higher than the 92.8% rate for indirect restorations made from resin-based composites. A temporal comparison of older versus newer studies indicated an improved outcome for indirect resin-based composite restorations over time, suggesting that advancements in the materials used for indirect resin-based composites may have led to enhanced performance.
Sailer et al. (2015)[33]	54	After a minimum observation period of three years, all-ceramic single-unit crowns were found to have survival rates comparable to those of metal-ceramic crowns. This equivalence in performance was specific to crowns constructed from leucite or lithium-disilicate reinforced glass ceramics or oxide ceramics. Crowns fabricated from densely sintered zirconia were found to possess a heightened risk of chipping of the veneering ceramic and issues with retention.
Sequeira-Byron et al. (2015)[34]	1	There is insufficient evidence to assess the effects of crowns compared with direct restorations for the restoration of root filled teeth.
Van den Breemer et. al (2015)[35]	20	The clinical studies included in the systematic review did not pinpoint a particular adhesive luting protocol as the optimal choice for posterior restorations made from glass-ceramic.
Grivas et al. (2014)[36]	14	There is insufficient evidence to answer whether any difference exist inlays between inlays made from resin-based composite, ceramic, or gold.<br>There is insufficient evidence to make recommendations for the use of indirect resin-based composite inlays over direct resin-based composite restorations regarding longevity. Most of the studies found that, in terms of esthetic quality and postoperative sensitivity, the differences between these two types of restorations were negligible. Restoration with resin-based composite inlays seems to be a better solution for premolars than for molars. The included studies could not conclusively determine the influence of cavity size on the clinical performance of indirect resin-based composite inlays.
Pieger et al. (2014)[37]	12	Short-term evidence (1 to 5 years) for lithium disilicate single-unit crowns shows an excellent survival rate, with a cumulative survival of 100% at two years and 97.8% at five years. However, medium-term survival data (5 to 10 years) is scant, with a single study indicating a 10-year cumulative survival rate of 96.7%. It was also observed that restoration failures occurred more commonly in posterior teeth compared with anterior teeth.
Fron Chabouis et al. (2013)[38]	2	Comparing inlays and onlays made from ceramic and resin-based composite, the 3-year overall failure risk ratio was 2 (confidence interval 0.4–10.6) in favor of ceramic inlays though not statistically significant. The reported clinical scores showed considerable heterogeneity between trials and could not be combined. There is very limited evidence that inlays made from ceramics perform better than inlays made from resin-based composites in the short term. This result may not be valid in the long term, and further trial-based data are needed.
Hmaidouch et al. (2013)[39]	5	Some all-ceramic crowns are as wear friendly as metal-ceramic crowns. Ceramic restorations should be sufficiently polished after any chairside occlusal

		adjustment to minimize the risk of roughened ceramic causing antagonist tooth wear.
Takeichi et al. (2013)[40]	19	The estimated annual failure percentage was 1.5% for porcelain-fused-to-zirconia and metal-ceramic single-unit crowns in posterior teeth. The scarcity of data from clinical trials prevented the drawing of definitive conclusions regarding their clinical survival rates.
Wang et al. (2012)[41]	37	Tooth-supported all-ceramic crowns exhibited a higher fracture rate in molars compared with premolars. The frequency of fractures of veneering ceramic displayed no significant difference between molars and premolars.
Pol et al. (2011)[42]	3	No significant difference was found regarding longevity between ceramic and other posterior restorations over assessment periods up to 1 year. Regarding postoperative sensitivity, no difference between ceramics and other restorative materials was observed. Ceramic materials demonstrate a color match on par with other esthetic materials, at least for a period of up to 57 months.
Wittneben et al (2009)*[43]	16	A systematic review encompassed data of 1,957 single-tooth restorations with a mean follow-up duration of 7.9 years, revealing an annual failure rate of 1.75% per 100 restoration years, with a five-year survival rate estimated at 91.6%. Long-term survival for CAD-CAM restorations from Cerec 1, 2, and Celay matched that of traditional methods. Studies on newer CAD-CAM systems beyond three years were not found.
Heintze et al. (2010)[44]	7	Adhesively luted IPS Empress crowns showed a lower fracture rate in premolars than in molars.
Pjetursson et al. (2007)[45]	34	After five years of follow-up, the survival rates of densely sintered alumina crowns at 94.9% and reinforced glass–ceramic crowns at 93.7% were comparable to the rates observed for metal-ceramic crowns when used in posterior teeth. When employed for premolars and molars, InCeram crowns and glass-ceramic crowns displayed lower survival rates of 90.4% and 84.4%, respectively.
Kaiser et al. (2006)†[46] and Wassermann et al. (2006)†[47]	21	The five-year survival rate of In-Ceram Alumina crowns was similar to that of conventional metal-ceramic crowns in posterior teeth.
Hayashi et al. (2003)*[48]	46	Over assessment periods of up to one year, no significant differences were found in longevity or postoperative sensitivity between ceramic restorations and other types of posterior restorations.
Martin et al. (1999)*[49]	15	The available data confirms that ceramic inlays created using the Cerec system are clinically successful, with an average survival rate of 97.4% across 4.2 years. Inlay fractures were the predominant reason for failure.

*Records identified through hand searching
†Reports based on the same systematic review
Abbreviations: *CAD-CAM,* computer-aided design and computer-aided manufacturing

REFERENCES

1. Bresser RA, Hofsteenge JW, Wieringa TH, et al. Clinical longevity of intracoronal restorations made of gold, lithium disilicate, leucite, and indirect resin composite: a systematic review and meta-analysis. *Clin Oral Investig*. 2023;27(9):4877–4896.
2. Lopes-Fatturi A, Wambier L, Rolim TZC, et al. Restorative techniques for permanent first molars affected by hypomineralization: a systematic review. *Pediatr Dent*. 2022;44(1):17–24.
3. Fathy H, Hamama HH, El-Wassefy N, et al. Clinical performance of resin-matrix ceramic partial coverage restorations: a systematic review. *Clin Oral Investig*. 2022;26(5):3807–3822.
4. Ferrari M, Ferrari Cagidiaco E, Pontoriero DIK, et al. Survival rates of endodontically treated posterior teeth restored with all-ceramic partial-coverage crowns: when systematic review fails. *Int J Environ Res Public Health*. 2022;19(4.
5. Hardan L, Mancino D, Bourgi R, et al. Treatment of tooth wear using direct or indirect restorations: a systematic review of clinical studies. *Bioengineering (Basel)*. 2022;9(8.
6. Leitão CIM, a Brito, Fern, et al. Clinical performance of monolithic CAD/CAM tooth-supported zirconia restorations: systematic review and meta-analysis. *J Prosthodont Res*. 2022;66(3):374–384.
7. McGrath CE, Bonsor SJ. Survival of direct resin composite onlays and indirect tooth-coloured adhesive onlays in posterior teeth: a systematic review. *Br Dent J*. 2022.
8. Tennert C, Suárez Machado L, Jaeggi T, et al. Posterior ceramic versus metal restorations: a systematic review and meta-analysis. *Dent Mater*. 2022;38(10):1623–1632.
9. Aziz A, el-Mowafy O, Paredes S. Clinical outcomes of lithium disilicate glass-ceramic crowns fabricated with CAD/CAM technology: a systematic review. *Dent Med Probl*. 2020;57(2):197–206.
10. Al-Dabbagh RA. Survival and success of endocrowns: a systematic review and meta-analysis. *J Prosthet Dent*. 2021;125(3):415.e1-415.e9.
11. Dioguardi M, Alovisi M, Troiano G, et al. Clinical outcome of bonded partial indirect posterior restorations on vital and non-vital teeth: a systematic review and meta-analysis. *Clin Oral Investig*. 2021;25(12):6597–6621.
12. Zou Y, Bai J, Xiang JZ. Survival rate of ceramic inlay and onlay restorations in posterior teeth with one-surface or multi-surface after a 10-year follow-up: a systematic review and meta-analysis. *Vojnosanitetski Pregled*. 2021;78(5):549–555.
13. Al-Haj Husain N, Özcan M, Molinero-Mourelle P, et al. Clinical performance of partial and full-coverage fixed dental restorations fabricated from hybrid polymer and ceramic CAD/CAM materials: a systematic review and meta-analysis. *J Clin Med*. 2020;9(7.
14. Biagioni, A, Pecciarini, et al. The role of occlusion on full mouth rehabilitation by adhesive partial crowns. A systematic review. *Journal of Osseointegration*. 2020;12(2):161–166.
15. Govare N, Contrepois M. Endocrowns: a systematic review. *J Prosthet Dent*. 2020;123(3):411-418.e9.
16. Komine F, Honda J, Kusaba K, et al. Clinical outcomes of single crown restorations fabricated with resin-based CAD/CAM materials. *J Oral Sci*. 2020;62(4):353–355.
17. Papia E, Habib W, Larsson C. The influence of different designs, materials and cements on the success and survival rate of endocrowns. A systematic review. *Eur J Prosthodont Restor Dent*. 2020;28(3):100–111.
18. Solá-Ruíz MF, Baima-Moscardó A, Selva-Otaolaurruchi E, et al. Wear in antagonist teeth produced by monolithic zirconia crowns: a systematic review and meta-analysis. *J Clin Med*. 2020;9(4.
19. Thomas RM, Kelly A, Tagiyeva N, et al. Comparing endocrown restorations on permanent molars and premolars: a systematic review and meta-analysis. *Br Dent J*. 2020.
20. Vetromilla BM, Opdam NJ, Leida FL, et al. Treatment options for large posterior restorations: a systematic review and network meta-analysis. *J Am Dent Assoc*. 2020;151(8):614-624.e18.
21. Gou M, Chen H, Kang J, et al. Antagonist enamel wear of tooth-supported monolithic zirconia posterior crowns in vivo: a systematic review. *J Prosthet Dent*. 2019;121(4):598–603.
22. Rodrigues SB, Franken P, Celeste RK, et al. CAD/CAM or conventional ceramic materials restorations longevity: a systematic review and meta-analysis. *J Prosthodont Res*. 2019;63(4):389–395.
23. Sampaio, Flávia B W R, Özcan M, Gimenez TC, et al. Effects of manufacturing methods on the survival rate of ceramic and indirect composite restorations: a systematic review and meta-analysis. *J Esthet Restor Dent*. 2019;31(6):561–571.

24. Abduo J, Sambrook RJ. Longevity of ceramic onlays: a systematic review. *J Esthet Restor Dent*. 2018;30(3):193–215.
25. Alves de Carvalho, Inês Filipa, Santos Marques TM, Araújo FM, et al. Clinical performance of CAD/CAM tooth-supported ceramic restorations: a systematic review. *Int J Periodontics Restorative Dent*. 2018;38(4):e68-e78.
26. Angeletaki F, Gkogkos A, Papazoglou E, et al. Direct versus indirect inlay/onlay composite restorations in posterior teeth. A systematic review and meta-analysis. *J Dent*. 2016;53):12–21.
27. Da Veiga AMA, Cunha AC, Ferreira, Daniele Masterson Tavares Pereira, et al. Longevity of direct and indirect resin composite restorations in permanent posterior teeth: a systematic review and meta-analysis. *J Dent*. 2016;54):1–12.
28. Morimoto S, Rebello de Sampaio, F B W, Braga MM, et al. Survival rate of resin and ceramic inlays, onlays, and overlays: a systematic review and meta-analysis. *J Dent Res*. 2016;95(9):985–994.
29. Kassardjian V, Varma S, Andiappan M, et al. A systematic review and meta analysis of the longevity of anterior and posterior all-ceramic crowns. *J Dent*. 2016;55):1–6.
30. Sedrez-Porto JA, Rosa, Wellington Luiz de Oliveira da, Da Silva AF, et al. Endocrown restorations: a systematic review and meta-analysis. *J Dent*. 2016;52):8–14.
31. Tsirogiannis P, Reissmann DR, Heydecke G. Evaluation of the marginal fit of single-unit, complete-coverage ceramic restorations fabricated after digital and conventional impressions: a systematic review and meta-analysis. *J Prosthet Dent*. 2016;116(3):328-335.e2.
32. Mangani F, Marini S, Barabanti N, et al. The success of indirect restorations in posterior teeth: a systematic review of the literature. *Minerva Stomatol*. 2015;64(5):231–240.
33. Sailer I, Makarov NA, (Keine Angabe), et al. All-ceramic or metal-ceramic tooth-supported fixed dental prostheses (FDPs)? A systematic review of the survival and complication rates. Part I: single crowns (SCs). *Dent Mater*. 2015;31(6):603–623.
34. Sequeira-Byron P, Fedorowicz Z, Carter B, et al. Single crowns versus conventional fillings for the restoration of root-filled teeth. *Cochrane Database Syst Rev*. 2015;2015(9):CD009109.
35. Van den Breemer CRG, Gresnigt MMM, Cune MS. Cementation of glass-ceramic posterior restorations: a systematic review. *Biomed Res Int*. 2015;2015):148954.
36. Grivas E, Roudsari RV, Satterthwaite JD. Composite inlays: a systematic review. *Eur J Prosthodont Restor Dent*. 2014;22(3):117–124.
37. Pieger S, Salman A, Bidra AS. Clinical outcomes of lithium disilicate single crowns and partial fixed dental prostheses: a systematic review. *J Prosthet Dent*. 2014;112(1):22–30.
38. Fron Chabouis H, Smail Faugeron V, Attal J-P. Clinical efficacy of composite versus ceramic inlays and onlays: a systematic review. *Dent Mater*. 2013;29(12):1209–1218.
39. Hmaidouch R, Weigl P. Tooth wear against ceramic crowns in posterior region: a systematic literature review. *Int J Oral Sci*. 2013;5(4):183–190.
40. Takeichi T, Katsoulis J, Blatz MB. Clinical outcome of single porcelain-fused-to-zirconium dioxide crowns: a systematic review. *J Prosthet Dent*. 2013;110(6):455–461.
41. Wang X, Fan D, Swain MV, et al. A systematic review of all-ceramic crowns: clinical fracture rates in relation to restored tooth type. *Int J Prosthodont*. 2012;25(5):441–450.
42. Pol CWP, Kalk W. A systematic review of ceramic inlays in posterior teeth: an update. *Int J Prosthodont*. 2011;24(6):566–575.
43. Wittneben J-G, Wright RF, Weber H-P, et al. A systematic review of the clinical performance of CAD/CAM single-tooth restorations. *Int J Prosthodont*. 2009;22(5):466–471.
44. Heintze SD, Rousson V. Fracture rates of IPS Empress all-ceramic crowns-a systematic review. *Int J Prosthodont*. 2010;23(2):129–133.
45. Pjetursson BE, Sailer I, Zwahlen M, et al. A systematic review of the survival and complication rates of all-ceramic and metal-ceramic reconstructions after an observation period of at least 3 years. Part I: single crowns. *Clin Oral Implants Res*. 2007;18):73–85.
46. Kaiser M, Wasserman A, Strub JR. Long-term clinical results of VITA In-Ceram Classic: a systematic review. *Schweiz Monatsschr Zahnmed*. 2006;116(2):120–128.
47. Wassermann A, Kaiser M, Strub JR. Clinical long-term results of VITA In-Ceram Classic crowns and fixed partial dentures: a systematic literature review. *Int J Prosthodont*. 2006;19(4):355–363.
48. Hayashi M, Wilson NHF, Yeung CA, et al. Systematic review of ceramic inlays. *Clin Oral Investig*. 2003;7(1):8–19.

49. Martin N, Jedynakiewicz NM. Clinical performance of CEREC ceramic inlays: a systematic review. *Dent Mater*. 1999;15(1):54–61.
